# Acute Neurologic Illness of Unknown Etiology in Children — Colorado, August–September 2014

**Published:** 2014-10-10

**Authors:** Daniel M. Pastula, Negar Aliabadi, Amber K. Haynes, Kevin Messacar, Teri Schreiner, John Maloney, Samuel R. Dominguez, Emily Spence Davizon, Eyal Leshem, Marc Fischer, W. Allan Nix, M. Steven Oberste, Jane Seward, Daniel Feikin, Lisa Miller

**Affiliations:** 1 Epidemic Intelligence Service, CDC; 2 Division of Viral Diseases, National Center for Immunization and Respiratory Diseases, CDC; 3 Children’s Hospital Colorado and University of Colorado School of Medicine; 4 Colorado Department of Public Health and Environment; 5 Division of Vector-Borne Diseases, National Center for Emerging and Zoonotic Infectious Diseases, CDC.

On September 12, 2014, CDC was notified by the Colorado Department of Public Health and Environment of a cluster of nine children evaluated at Children’s Hospital Colorado with acute neurologic illness characterized by extremity weakness, cranial nerve dysfunction (e.g., diplopia, facial droop, dysphagia, or dysarthria), or both. Neurologic illness onsets occurred during August 8–September 15, 2014. The median age of the children was 8 years (range = 1–18 years). Other than neck, back, or extremity pain in some patients, all had normal sensation. All had a preceding febrile illness, most with upper respiratory symptoms, occurring 3–16 days (median = 7 days) before onset of neurologic illness. Seven of eight patients with magnetic resonance imaging of the spinal cord had nonenhancing lesions of the gray matter of the spinal cord spanning multiple levels, and seven of nine with magnetic resonance imaging of the brain had nonenhancing brainstem lesions (most commonly the dorsal pons). Two of five with magnetic resonance imaging of the lumbosacral region had gadolinium enhancement of the ventral nerve roots of the cauda equina. Eight children were up to date on polio vaccination. Eight have not yet fully recovered neurologically.

Eight patients demonstrated a mild to moderate cerebrospinal fluid (CSF) pleocytosis (>5 white blood cells/*μ*L), predominantly lymphocytic, consistent with an inflammatory or infectious process. CSF glucose was normal; CSF protein was normal or mildly elevated. Initial testing of CSF from eight patients showed no evidence of West Nile virus antibodies, although further testing is pending. CSF testing for enteroviruses, including enterovirus D68 (EV-D68), enterovirus 71, and poliovirus, by reverse transcription–polymerase chain reaction (RT-PCR) was negative in all patients. Other CSF tests for infectious causes were unrevealing.

Initial nasopharyngeal specimens were available for eight children. Six were positive for rhinovirus/enterovirus by RT-PCR. These six positive nasopharyngeal specimens were subsequently typed: four were identified as EV-D68, one as rhinovirus A24, and one was not EV-D68 but has not been typed further. The specimen positive for rhinovirus A24 also was positive for adenovirus by RT-PCR. Single rectal swabs or stool samples from eight patients were negative for enterovirus (including poliovirus) by RT-PCR.

This cluster of acute neurologic illnesses occurred against a backdrop of detection of EV-D68 causing severe respiratory disease in many parts of the United States, including Colorado (1,2). There are two case reports in the literature of EV-D68 causing neurologic illness (acute flaccid paralysis and encephalomyelitis) as evidenced by detection of EV-D68 in the CSF (3,4). However, given the current suspected widespread circulation of EV-D68 respiratory infections in Colorado, and the antecedent respiratory illness in most of these children, the detection of EV-D68 in nonsterile upper respiratory tract specimens in those with neurologic illness might be coincidental. Epidemiologic and laboratory investigations of these cases are ongoing.

On September 19, the Colorado Department of Public Health and Environment issued a Health Alert informing Colorado clinicians of this cluster and requesting reports of similar cases. One additional case with similar neurologic findings was reported as a result of this advisory and is currently under investigation. On September 26, CDC issued a national Health Advisory (available at http://www.bt.cdc.gov/han/han00370.asp ), which provides guidance for identifying and reporting cases. Clinicians should report to their local and state health departments patients aged ≤21 years with 1) acute onset of focal limb weakness occurring on or after August 1, 2014, and 2) magnetic resonance imaging showing a spinal cord lesion largely restricted to gray matter. To prevent infections in general, persons should stay home if they are ill, wash their hands often with soap and water, avoid close contact (such as touching and shaking hands) with those who are ill, and clean and disinfect frequently touched surfaces.
